# Risk analysis and prediction of visceral leishmaniasis dispersion in São Paulo State, Brazil

**DOI:** 10.1371/journal.pntd.0005353

**Published:** 2017-02-06

**Authors:** Anaiá da Paixão Sevá, Liang Mao, Fredy Galvis-Ovallos, Joanna Marie Tucker Lima, Denis Valle

**Affiliations:** 1 Department of Mathematics, University of Florida, Gainesville, Florida, United States of America; 2 Department of Preventive Veterinary Medicine and Animal Health, School of Veterinary Medicine and Animal Sciences, University of São Paulo, São Paulo, Brazil; 3 Department of Geography, University of Florida, Gainesville, Florida, United States of America; 4 Department of Epidemiology, School of Public Health, University of São Paulo, São Paulo, Brazil; 5 School of Forest Resources and Conservation, University of Florida, Gainesville, Florida, United States of America; Hospital Universitário Professor Edgard Santos, BRAZIL

## Abstract

Visceral leishmaniasis (VL) is an important neglected disease caused by a protozoan parasite, and represents a serious public health problem in many parts of the world. It is zoonotic in Europe and Latin America, where infected dogs constitute the main domestic reservoir for the parasite and play a key role in VL transmission to humans. In Brazil this disease is caused by the protozoan *Leishmania infantum chagasi*, and is transmitted by the sand fly *Lutzomyia longipalpis*. Despite programs aimed at eliminating infection sources, the disease continues to spread throughout the Country. VL in São Paulo State, Brazil, first appeared in the northwestern region, spreading in a southeasterly direction over time. We integrate data on the VL vector, infected dogs and infected human dispersion from 1999 to 2013 through an innovative spatial temporal Bayesian model in conjunction with geographic information system. This model is used to infer the drivers of the invasion process and predict the future progression of VL through the State. We found that vector dispersion was influenced by vector presence in nearby municipalities at the previous time step, proximity to the Bolívia-Brazil gas pipeline, and high temperatures (i.e., annual average between 20 and 23°C). Key factors affecting infected dog dispersion included proximity to the Marechal Rondon Highway, high temperatures, and presence of the competent vector within the same municipality. Finally, vector presence, presence of infected dogs, and rainfall (approx. 270 to 540mm/year) drove the dispersion of human VL cases. Surprisingly, economic factors exhibited no noticeable influence on disease dispersion. Based on these drivers and stochastic simulations, we identified which municipalities are most likely to be invaded by vectors and infected hosts in the future. Prioritizing prevention and control strategies within the identified municipalities may help halt the spread of VL while reducing monitoring costs. Our results contribute important knowledge to public and animal health policy planning, and suggest that prevention and control strategies should focus on vector control and on blocking contact between vectors and hosts in the priority areas identified to be at risk.

## Author summary

Visceral leishmaniasis [VL] is an important but neglected tropical disease that occurs worldwide. In areas where the disease is zoonotic, it is considered a serious public and animal health problem. In Brazil, despite the existing prevention and control programs, the disease is spreading, and in São Paulo State its dispersion presents a distinct temporal-geographic pattern. The goal of our study was to understand the landscape, climatic and economic factors that influence the dispersion of visceral leishmaniasis in Sao Paulo State, and then use these findings to predict its spread over time and space. To this end, we integrated data on the sand fly vector of the causative parasitic agent of VL, infected dogs and human cases. We find that landscape and climate were more important than economic factors in predicting competent vector, infected human and infected dog distributions, and the presence of the competent vector and infected dogs strongly influenced the dispersion of infected humans. Our study represents the first integrated investigation of vector and infected host invasion potential for individual municipalities, contributing to VL disease prevention and control planning in São Paulo State.

## Introduction

Visceral leishmaniasis (VL), also known as kala-azar, is characterized by irregular bouts of fever, weight loss, enlargement of the spleen and liver, and anemia [1]. It is an important disease that occurs around the world [2], and Brazil is one of six countries that concentrates 90% of new human VL cases [1]. In Europe and Latin America, the disease is zoonotic, and in Brazil it is caused by the parasite *Leishmania infantum chagasi (L*. *i*. *chagasi)*, which is transmitted to humans and other animals (such as rodents and wild and domestic dogs) mainly through the bites of a sand fly (*Lutzomyia longipalpis*) vector [3]. Despite being historically known as a rural endemic disease, VL has reached endemic and epidemic proportions in many large Brazilian cities since the 1980s [3,4,5], representing a serious public health problem [2].

Where the disease is zoonotic, infected dogs constitute the main domestic parasite reservoir and play a key role in VL transmission to humans [6,7]. Hence, one of the main VL prevention and control strategies adopted by the Brazilian government has been to test and cull infected dogs [8]. Despite these efforts, ongoing program initiatives to combat infection sources have failed to reduce disease incidence in humans [9]. On the contrary, the number of new notified cases has kept rising and the disease is spreading across Brazil [10,11]].

The Brazilian State of São Paulo has experienced rapid spread of human and dog VL over the last two decades, mainly associated with the expansion of the vector’s geographic range. In 1998, the sand fly that transmits VL was found in only two municipalities, but since then it has been recorded in 164 municipalities by 2014, principally in the western part of the State [12]. In 1998 and 1999 the first infected dog [13] and human cases were identified, respectively, in municipalities where the vector had already been confirmed [14]. These changes coincided with the construction of the Bolívia-Brazil gas pipeline, which recruited large numbers of migrant workers from various VL endemic regions (e.g., Mato Grosso do Sul). The pipeline was built across São Paulo State between 1997 and 1999, and within ten years, 53 São Paulo municipalities confirmed 954 human cases of VL and reported 81 associated deaths between 2010 and 2015 [10,15].

The expansion of VL in São Paulo occurred along a major axis extending from the northwest to the southeast towards the Bauru region, following both the Bolivia-Brazil gas pipeline and the Marechal Rondon Highway [16]. Migrant workers arrived during the construction of the gas pipeline by way of the Marechal Rondon Highway, which crosses from the northwest corner of the state to the state capital in the southeast. Other factors may also have contributed to VL expansion, such as socioeconomic conditions, climatic factors and ecological imbalances that influence vectors responsible for transmitting the *L*. *i*. *chagasi* parasite. For instance, Sundar et al. [17] found an association between poverty and VL incidence in humans, and suggested that environmental factors created by poor housing conditions encourages sand fly breeding. Nevertheless, the ultimate factors that contribute to the spread of VL remain uncertain.

Infectious disease outbreaks have both spatial and temporal dimensions [18]. Spatial heterogeneities may arise due to differences in the distribution of vectors and other risk factors, different patterns of intra-urban vector-host contact, and variations in population susceptibility [5]. Few studies have assessed the spatio-temporal distribution, including underlying risk factors, of VL [19–22], particularly in Brazil [23–25]. Furthermore, to the best of our knowledge, this is the first study to predict the spread of VL through time and space in São Paulo State, where the disease now seems to have reached the capital. Unlike other studies [23,24], we employ an innovative spatio-temporal Bayesian model and GIS (Geographical Information Systems) to infer factors driving the invasion of VL by integrating data on hosts and vector locations between 1999 and 2013 with disease natural history. This Bayesian model is then used to predict the epidemic’s future geographical progression. Prediction maps identify strategic intervention areas for controlling vector and infected host dispersion, and provide valuable information to public health authorities to help spatially optimize VL prevention and control measures.

## Methodology

### Municipalities

**Fig 1:**
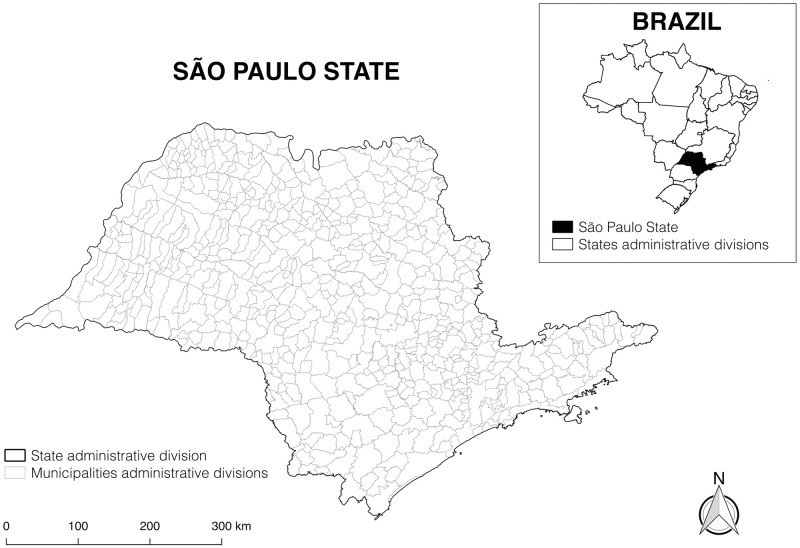
Brazil and São Paulo State.

São Paulo State is comprised of 645 municipalities (Fig 1), with geographic areas ranging from 118,951 and 1,977,951 km^2^. Based on digitized municipal boundary maps produced by the Brazilian Institute of Geography and Statistics [26], we calculated geographic centroids for each Municipality using QGIS (version 2.8), which are then used in subsequent analyses.

### Vector presence and infected hosts

For each Municipality, we obtained data on the presence and absence of (1) VL sand fly vectors, (2) autochthonous VL human cases, and (3) VL seropositive dogs, from 1999 to 2013. Data on the distribution of the vector *Lu*. *longipalpis* were obtained from the São Paulo State Secretary of Health, as described by Casanova et al. [12]. The notification of human VL cases is compulsory throughout Brazil, and is monitored through (1) spontaneous reporting to the Brazilian Health Units, (2) active search for cases in known transmission areas, (3) home visits by health professionals, and (4) patient referrals through the primary health care network [27]. We assume these records to be the best available for the study area. They are freely available through the Brazilian Ministry of Health website [10,11,15,28]. Data on infected dog cases were obtained from canine surveys carried out by municipal governments, the Adolfo Lutz Institute, and the Secretary of Health of São Paulo State. Municipalities are also expected to notify the first-confirmed occurrence of *L*. *i*. *chagasi* in dogs. Data from municipalities arise from dogs suspected to be infected that are subsequently evaluated using a parasitological test.

### Covariates

To investigate factors driving the spread of sand fly vectors, VL infected dogs and infected human dispersion across São Paulo State, we examined the following covariates at the municipal level: invasion pressure, climatic features, economic factors, and the distance between each municipality’s centroid and (1) the Marechal Rondon Highway and (2) the Bolivia-Brazil gas pipeline.

#### Invasion pressure

To account for the spatial contagion of the vectors, infected dogs and humans, we created an invasion pressure index for each population and each municipality that takes into account the spatial configuration of already invaded municipalities. More specifically, the invasion pressure index for municipality *i* at time *t* was defined as:

$$P_{it}=\underset{j}{\sum }w_{ij}\times y_{j\left(t-1\right)}$$

where *w*_*ij*_ are distance-decay weights and *y*_*j*(*t−*1)_ is the invasion status (0 = not invaded, 1 = invaded) of municipality *j* at time *t*-1. Weights are inversely proportional to the *D*_*ij*_ (the Euclidean distance between the geographic centroids of two municipalities *i* and *j*), formulated as $w_{ij}=\frac{\frac{1}{D_{ij}}}{\sum_{j}\frac{1}{D_{ij}}}$.

#### Climate and topography

We included annual average temperature (*temp*), rainfall (*rain*), and altitude (*alt*) data for each municipality in the model. These data were originally compiled from multiple sources, summarized and made publicly available in Alvares et al. [29]. Altitude data were originally available in raster format with a spatial resolution of 90m [30] and were subsequently aggregated to the municipal level by averaging all cells within each municipality. Both rainfall and temperature databases were obtained from weather stations monitored by the Food and Agriculture Organization of the United Nations (FAO/ONU) [30].

#### Economy

Data on São Paulo municipalities’ Annual Gross Domestic Product (*GDP*) were only available for part of the study period spanned by the VL vector and host data (i.e., 2000 to 2012; [31]). For this reason, these data were averaged for each municipality (S1 Fig).

#### The gas pipeline and the highway

Location data for the Bolivia-Brazil gas pipeline and Marechal Rondon Highway were obtained from the Brazilian Transport Ministry [32] and the Brazilian National Department of Transport Infrastructure [33], respectively (S2 Fig). Euclidean distances were calculated from each Municipality’s centroid to the pipeline and the highway.

### Model description

Bayesian models for disease mapping have primarily utilized a generalized linear model framework, with regression parameters modeled as random effects with spatial (or space-time) covariance matrix [34]. Specific studies that applied these models to leishmaniasis in Brazil include Karagiannis-Voules et al. [23] and Assunção et al. [24]. The Bayesian model we developed to predict spatio-temporal spread of VL in São Paulo State differs substantially from these earlier approaches in that our model integrates data on vectors, infected dogs and humans, and accounts for spatial and temporal correlation through a spatially weighted infection pressure index, as described below.

Our observations consist of *y*_*it*_, which is a binary outcome indicating whether municipality *i* was invaded in year *t*, where *i* = 1,…,645, and *t* = 1,…,15. The time subscript *t* corresponds to annual time steps from 1999 to 2013. In all of our models, we assumed that

$$y_{it}\sim Bernoulli\left(\theta_{it}\right)$$

where the probability of invasion is given by *θ*_*it*_ If a municipality has already been invaded by a competent vector (or by an infected dog or human), we assume it will keep its invasion status throughout the study period. This assumption can be stated as:

$$p\left(y_{it}=1|y_{i\left(t-1\right)}=1\right)=\theta_{it}=1$$

If the municipality has not been previously invaded (i.e., *y*_*i*(*t*-1)_ = 0), we assume that:

$$p\left(y_{it}=1|y_{i\left(t-1\right)}=0\right)=\theta_{it}=\frac{\text{exp}\left(x_{it}^{T}\beta \right)}{1+\text{exp}\left(x_{it}^{T}\beta \right)}$$

where $x_{it}^{T}$ and ***β*** are vectors of covariates and regression parameters, respectively.

We base our investigations of municipality “invasion” on the following premises, related to the competent vector and infected hosts:

1. The competent vector dispersion does not depend on the presence of infected hosts. Therefore, we assume that the probability of vector invasion for municipality *i* at time *t* $\left(\theta_{it}^{v}\right)$ (assuming this municipality had not yet been invaded) is given by:
  $$\;logit\left(\theta_{it}^{v}\right)=\;\beta_{0}+\;\beta_{1}P_{it}^{v}+\;\beta_{2}gas_{i}+\;\beta_{3}rod_{i}+\beta_{4}\text{log}\left(gdp_{i}\right)+\beta_{5}alt_{i}+\beta_{6}temp_{i}+\;\beta_{7}\text{log}\left(rain_{i}\right)$$
2. Dogs can become infected by the vector at the focal municipality (Municipality *i*) or they may be infected elsewhere and move into the focal municipality. Therefore, we assume that the probability of invasion by infected dogs in Municipality *i* at time *t* $\theta_{it}^{d}$ (assuming this municipality had not yet been invaded) depends on the presence of the vector *vect*_*it*_ as well as the other covariates:
  $$logit\left(\theta_{it}^{d}\right)=\;\beta_{0}+\;\beta_{1}P_{it}^{d}+\beta_{2}gas_{i}+\beta_{3}rod_{i}+\;\beta_{4}\text{log}\left(gdp_{i}\right)\;+\;\beta_{5}alt_{i}+\beta_{6}temp_{i}+\;\beta_{7}\text{log}\left(rain_{i}\right)+\;\beta_{8}vect_{it}$$
3. Infection in humans depends on vector presence, and when dogs and vectors occupy the same space, the disease becomes established [35,36]. Thus, we assume that the probability of invasion by infected humans in a municipality *i* at time *t* $\theta_{it}^{h}$ (assuming this municipality had not yet been invaded) depends on the presence of the vector *vect*_*it*_ and the presence of infected dogs *dog*_*it*_ as well as the other covariates:
  $$\begin{array}{l} logit\left(\theta_{it}^{h}\right)=\beta_{0}+\beta_{1}P_{it}^{h}+\;\beta_{2}gas_{i}+\;\beta_{3}rod_{i}+\beta_{4}\text{log}\left(gdp_{i}\right)+\beta_{5}alt_{i}\;+\beta_{6}temp_{i}\\ +\;\beta_{7}\text{log}\left(rain_{i}\right)+\beta_{8}vect_{it}+\beta_{9}dog_{it}+\beta_{10}\left(vect_{it}*dog_{it}\right) \end{array}$$

Finally, we assume no false positives or false negatives. The models formulated above can be thought of as generalizations of the geometric distribution (number of trials before the first success/invasion), where the individual success/invasion probabilities are not constant and spatial and temporal dependence are taken into account by evaluating the status of other municipalities in the previous time step, as captured by the invasion pressure covariate for vectors, infected dogs and infected humans ($P_{it}^{v}$, $P_{it}^{d}$, $P_{it}^{h}$ respectively). Our analysis is conditional on the status of each municipality in year 1. Thus, the likelihood of this model is given by:

$$p\left(y_{.2},\ldots ,y_{.T}|X,\beta ,y_{.1}\right)\propto \overset{I}{\underset{i=1}{\prod }}\overset{T}{\underset{t=2}{\prod }}p\left(y_{it}=1|y_{.\left(t-1\right)},x_{it},\beta \right)$$

We fit this model in a Bayesian framework using JAGS, which enables us to coherently represent uncertainty when creating predictions of invasion probability from 2014 to 2020. Our Bayesian model is likely to be very useful for researchers interested in understanding disease spread, and for this reason, we include a detailed tutorial-style appendix that describes how to implement the model (S1 Appendix).

### Predictions

The model described above was also used to predict the “invasion” of VL-free municipalities from 2014 to 2020 by using the posterior distribution of the parameters and forward simulation of the invasion process. For each sample of the posterior distribution, we sequentially created a forward simulation for the vector, for the infected dogs, and finally for infected humans, using 2013 as the starting point. We then summarized these synthetic invasion scenarios by calculating the probability that each municipality has been invaded. Our annual predictions of these invasion probabilities were mapped (using QGIS version 2.8), and maps were compiled into a time-loop video (available online) that incorporates both actual vector and infected host presence/absence data from 1999–2013 as well as predicted probability of invasion from 2014–2020.

### Model validation

For model validation, we obtained 2015 data on municipalities invaded by the vector and VL infected humans. Model validation was accomplished by comparing the 2015 predicted invasion probabilities with actual 2015 observations. Although only five and 15 new municipalities were invaded by infected humans or vectors in 2015, respectively, these data were still useful in evaluating the model’s predictive power.

## Results

### Risk factors

A summary of our invasion risk factor analysis for vectors, dogs and humans is given in Table 1. Statistically significant covariates for vector dispersion included the invasion pressure index (which takes into account vector presence in nearby municipalities during the previous time step), proximity to the gas pipeline, and temperature. Covariates that significantly affected the dispersion of infected dogs were proximity to Highway, temperature, and presence of the competent vector within the same Municipality. Finally, the covariates that exhibited significant influence on the dispersion of infected humans were vector presence and the interaction term between vectors and infected dogs. The effect of rainfall was also marginally significant for human cases of VL.

**Table 1:** Parameter estimates for VL vector (*Lu*. *longipalpis*), VL infected dogs and VL infected humans.

Param.	Covariate	VECTOR		DOGS		HUMANS	
		Means (CI 95%)					
b0		-5.46	(-5.83/-5.12)	-6.79	(-7.41/-6.25)	-7.21	(-8.03/-6.551)
b1	Invasion pressure	5.51*	(4.11/6.89)	0.11	(-1.58/1.87)	0.19	(-1.51/1.870)
b2	Gas pipeline	-0.67*	(-1.01/-0.34)	-0.17	(-0.66/0.32)	-0.41	(-0.99/0.147)
b3	Highway	-0.25	(-0.65/0.14)	-1.02*	(-1.56/-0.51)	-0.14	(-0.81/0.49)
b4	GDP	-0.13	(-0.34/0.06)	-0.10	(-0.39/0.17)	-0.30	(-0.66/0.047)
b5	Altitude	-0.24	(-0.85/0.41)	0.56	(-0.34/1.45)	-0.22	(-1.40/1.013)
b6	Temperature	0.86*	(0.25/1.46)	1.54*	(0.67/2.44)	0.35	(-0.67/1.354)
b7	Rainfall	-0.28	(-0.80/0.12)	0.40	(-0.15/0.86)	-0.94*	(-1.92/-0.084)
b8	Vector	---	---	3.69*	(3.1/4.32)	1.45*	(0.52/2.303)
b9	Dog	---	---	---	---	1.18	(-0.35/2.563)
b10	Vector*Dog	---	---	---	---	1.46*	(0.03/2.973)

Param.: parameter symbols. GDP: Gross Domestic Product. 95% credible intervals (CI) are given between parentheses.

*Statistically significant slope parameters (i.e., parameters for which the 95% credible interval range did not include zero). Negative parameter estimates indicate a negative association between the covariate and the probability of invasion (e.g., greater distance to the gas pipeline decreases the invasion probability for vectors). Positive parameter estimates indicate a positive association between the covariate and the probability of invasion (e.g., higher temperatures increase the invasion probability by infected dogs).

Both vector and infected dog dispersions were strongly influenced by temperature. On the other hand, none of the dispersion patterns were affected by altitude variations or low economic productivity (GDP). Interestingly, results regarding proximity to the gas pipeline and the highway were inconsistent, suggesting potentially different dispersion mechanisms for vectors versus dogs.

### Predictions

**Fig 2:**
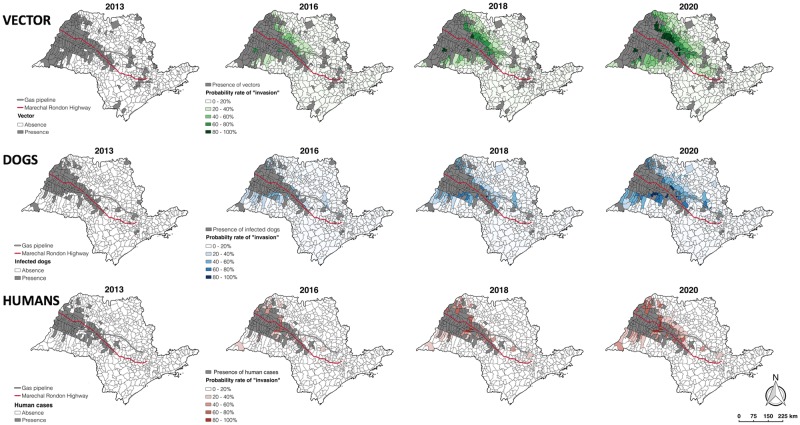
Actual (2013) and predicted (2016, 2018, 2020) spatial distribution of municipalities with presence of visceral leishmaniasis vector (*Lu*. *longipalpis* sand fly), infected dogs and infected humans in São Paulo State, Brazil. The probability of “invasion” was calculated using the posterior distribution of the parameters (described in the section “Covariates”) from the Bayesian model and forward simulations, as described in the section “Predictions”.

Maps of vector, infected dog, and infected human presence/absence for 2013 and prediction maps for 2016, 2018, and 2020 displaying “invasion” probabilities are shown in Fig 2. The effect of distance to the Highway on infected hosts is particularly evident in Fig 2. Vectors are spreading toward the north and east where municipalities intersect the gas pipeline, and therefore, these regions are associated with a higher probability of vector invasion (Fig 2 and Table 1). As a result, both infected dog and human invasion probabilities in municipalities crossed by the gas pipeline are also slightly higher, due to the influence of the vector in their dispersion (Table 1).

**Fig 3:**
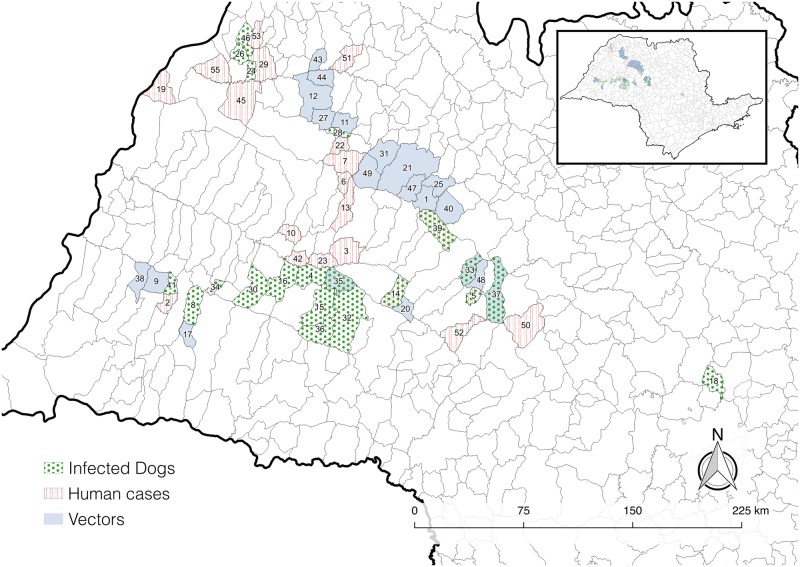
Spatial distribution of the top 20 municipalities in terms of 2020 invasion probability for vectors, infected dogs, and infected humans. Numbers assigned to municipalities are described in the S1 Table, as superscripts.

The majority of municipalities currently affected or predicted to be affected by vectors or hosts lie in the western part of São Paulo State. The top 20 municipalities with the highest invasion probabilities for vectors, infected dogs and infected humans are displayed in Fig 3 and listed in S1 Table. As expected for infected dogs, municipalities with higher invasion probabilities in 2020 were typically those already invaded by the vector (Fig 2). Three municipalities (enumerated as 33, 35 and 37 in Fig 3) have both vectors and infected dogs, and no infected humans (at least until 2013) (see in Fig 2). For infected humans, municipalities with the highest invasion probabilities in 2020 were those already invaded by both vectors and infected dogs. Three municipalities (enumerated as 5, 14 and 34 in Fig 3) have both infected dogs and humans, as well as the vector (see in Fig 2), which “arrived” before 2013.

### Model validation

**Fig 4:**
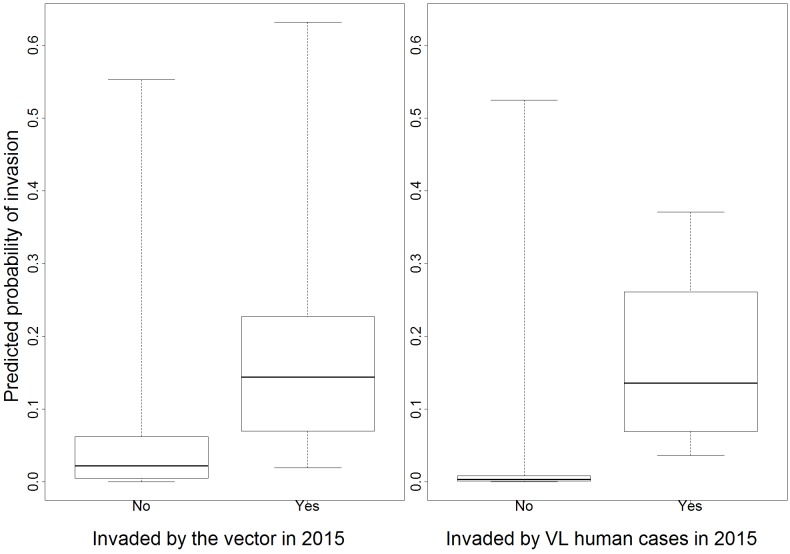
Predicted probability of invasion by vectors and human cases in São Paulo State municipalities by 2015 (y-axis) for invaded and not-invaded municipalities in 2015 (x-axis). Boxes represent the 25% and 75% percentiles, with the central horizontal line representing the median, while whiskers extend to the minimum and maximum values.

As expected, our models tended to assign higher invasion probabilities to “invaded” municipalities than to “non-invaded” municipalities, indicated by the taller boxes for the “invaded” municipalities in Fig 4. Furthermore, the difference in predicted probabilities for the invaded and non-invaded municipalities was greater for VL human cases than for vector presence (Fig 4), suggesting a higher predictive skill of our models for VL human cases rather than vector presence. This is likely due to the fact that vector and infected dog presence in 2013 is highly predictive of VL human case invasion in 2015. Indeed, two out of five municipalities invaded with VL human cases in 2015 were already invaded by infected dogs by 2013, and all five municipalities had already been invaded by the vector in 2013.

## Discussion

One of the most important risk factors for VL around the world is the migration of people from endemic to non-endemic regions [37]. During the construction of the Bolivia-Brazil gas pipeline, which began in 1998, thousands of workers moved from the city of Corumbá (an endemic area) to other cities and regions of Mato Grosso do Sul and neighboring states (all non-endemic areas), explaining the spatial pattern of disease establishment in this region [38,39,12]. Cardin et al. [16] were the first to observe a similar pattern in São Paulo State, finding an association between leishmaniasis and proximity to the Marechal Rondon Highway. They reasoned that the road served as an important conduit for arrival of migrants into São Paulo, connecting endemic and non-endemic regions [16]. The Marechal Rondon Highway provided such a link from western municipalities in the State to the capital city.

We found a significant influence of the gas pipeline on VL distribution, specifically on vector dispersion, and an effect of the Highway on the dispersion of infected dogs (see Table 1; note that the two coefficients are negative, suggesting that the smaller the distance, the greater the probability of invasion). While VL presence may have been initially influenced by the gas pipeline construction, it may now be spreading along major transport routes such as the Highway, as observed in our predictions (Fig 2). In their study of a leishmaniasis outbreak in Spain, Ruiz et al. suggested that construction of a dense road network created ideal conditions for the development of suitable habitats for both VL vectors and vector reservoirs [21]. In the same way, the Marechal Rondon Highway's presence in São Paulo altered environmental conditions by removing the native vegetation, and likewise, the construction of the gas pipeline may have prompted an increase of VL incidence by altering vector habitat.

We found no significant effect of altitude on vector or infected host dispersion in São Paulo State. Vector and infected hosts occurred in municipalities with elevation between 274m and 804m, but preferentially between 274m and 539m, representing 85.3% and 40% of the municipalities altitudes at the State, respectively (Fig 2 and S3 Fig). The vector itself was present in just a few municipalities between 804 and 1040m. In Belo Horizonte, Brazil, Etelvina et al. [40] also observed no influence of altitude in human VL cases; however, other studies conducted in the same area suggested a concentration of infected dogs and human VL cases between 780 and 880 m [41], or between 751m and 850m of altitude [42], and found that the majority of sand fly vectors occur at altitudes below 851m [42].

Factors such as temperature, relative humidity, and rainfall can influence sand fly population density [43]. In the present study we observed that higher temperatures (annual average between 19 and 23°C), occurring in the western part of the State (S4 Fig), favor the spread of vectors (Table 1). Furthermore, in our predictions (Fig 2), neither vectors nor infected hosts reached areas cooler than 17°C, typical of municipalities on the eastern side of the State (S4 Fig). Other studies revealed that temperature strongly influences the distributions of competent VL vectors and infected dogs in Brazilian endemic areas [44,45], as well as in parts of Europe [46,47]. In studies conducted over smaller spatial scales, where temperatures vary little throughout the year, temperature was found to have no effect on vector dispersion [48,49]. Temperature regulates several sand fly biological parameters [50]. In particular, low temperatures reduce the sand fly metabolism (thus increasing its longevity) [51,52] and biting rate, and also increase extrinsic incubation period, thereby negatively affecting the sandflies’ basic reproduction rate [50]. Hence, climate change, especially an increase in temperature, may accelerate the rate of geographical expansion of VL.

A map of average rainfall (S5 Fig) shows that dry areas (annual average as 96-119mm/year) predominate in the western part of São Paulo State, representing 69.3% (447/645) of all municipalities. Between 1998 and 2013, vectors and infected dogs were concentrated mainly in these western municipalities, and rainfall had no significant effect on their distributions (Table 1). On the other hand, rainfall affects the infected human dispersion (see Table 1; the negative coefficient suggests that lower rainfall increases the probability of invasion), and 89% of the municipalities with human VL cases experience an average rainfall of 96-109mm/year, and represent 42.8% (276/645) of all São Paulo municipalities. Studies suggest that dry seasons are associated with increased numbers of VL canine cases [53] as well as greater vector densities in Mato Grosso do Sul-Brazil [53,54] and Costa Rica [55]. In Spain, Pérez-Cutillas et al. [56] observed that areas with high human VL prevalence had significantly less rainfall during the sand fly season and more rainfall in wintertime. In addition, these authors suggested that greater rainfall during the coldest months of the year may increase survival of eggs and diapausing sand fly instars, while greater precipitation during the summer months may hamper adult sand fly flight activity.

In Brazil, other environmental factors have been positively correlated with anti-*Leishmania* antibodies in humans, including houses with mud floors and/or mud walls, lack of garbage collection, vegetation type, and presence of dogs, birds, or donkeys/horses in the neighborhood [57], as well as inadequate sanitation infrastructure [58]. These factors likely reflect an overlap of poorer housing with other unmeasured effects of poverty and/or an environment conducive to sand fly breeding [57]. High indices of vector diseases are often correlated with the world’s poorest regions, and low socioeconomic status of dog owners is associated with *L*. *i*. *chagasi* canine infection [35,59–63]. Lima et al. observed that *L*. *i*. *chagasi* infections in dogs and humans were intimately connected to environmental conditions associated with low incomes [57]. Karagiannis-Voules et al. found a direct association between socioeconomic factors and the incidence of human VL cases in Brazil [64]. In the present study, however, vector presence and infected host dispersions presented no significant relationship with income as measured by GDP. GDP varied only slightly among São Paulo municipalities where vectors and infected hosts were found (Fig 2), which may explain why we found no association. More stark differences in economic productivity (GDP) are evident in the eastern region of the State, but this region has displayed very low VL invasion up till now (Fig 2). Finally, better poverty metrics might present a stronger correlation with dispersion probability of vectors and infected hosts.

We originally hypothesized that the invasion pressure index would play a key role in dispersion of vectors, infected dogs and infected humans, but our results only showed an impact on vector dispersion, suggesting that spatial contagion is a key process only for vectors. The spatial contagion (as captured by our invasion pressure covariate) was not significant, given the presence of the vector, likely due to the relatively high mobility of dogs and humans, as compared to the vector. These results highlight the importance of simultaneously accounting for vectors and multiple hosts when assessing the spatial and temporal distribution of human VL cases. Presence of the vector itself is a poor predictor of disease transmission, because infected reservoirs or/and vectors must be present to establish parasite circulation in transmission foci [65]. Nonetheless, spatially targeted interventions aimed at eliminating the vector could be highly effective in halting vector dispersion, whereas similar spatial strategies targeting infected dogs or humans are less likely to succeed. Prioritizing insecticide spraying of residual action and other strategies that aim to eliminate or avoid VL vectors in municipalities with a high invasion probabilities represent an integral and necessary component of preventative measures for this disease.

In Brazil some studies found that the occurrence of VL in humans is associated with the presence of infected dogs in the same area [25,26,49,50,66,67]. Werneck et al. observed that increasing prevalence of canine infection effectively forecast high incidence of human VL, as did high prevalence of canine infection before and during an epidemic [60], and Lima et al. report a high likelihood of geographic intersection between seropositive humans and dogs [57]. In the present study, infected dogs also affect the dispersion of infected humans, but only when the competent vector is also present in the same environment. This particular dynamic occurs where the disease presents a zoonotic pattern, with dogs acting as the main reservoir.

Considering this zoonotic scenario, and in an effort to reduce or even eliminate human cases of VL, the Brazilian Leishmaniasis Control Program treats human cases and invests in the reduction of vector densities (spraying insecticide) and canine control (culling seropositive dogs) [27]. Nevertheless, these strategies, practiced since 1950, have met with little success in reducing VL incidence in humans. Instead, the number of human VL cases continues to rise, and the disease is spreading geographically becoming a serious public health problem [9]. Because the presence of the vector and infected dogs together affected the dispersion of human VL cases, we argue that while control strategies should continue to focus on vector-dog contact, inclusion of other measures that have demonstrated success in controlling the disease should also be promoted, such as dog vaccines [68,69] insecticide-impregnated collars [13,70–73] for a large percentage of dog population [74], application of insecticide of residual action in the environment, topically applied insecticide, and testing dogs before their introduction into non-endemic areas [75].

Other models used to predict zoonotic VL [19,23,65] do not integrate data on the vector and the two hosts (i.e., dogs and humans). Furthermore, unlike these other studies, we map annual probabilistic predictions for the vector, infected dogs and infected humans using GIS. Along with these innovations, we acknowledge important limitations regarding the data used in our statistical analysis. First, leishmaniasis cases are often under-reported in Brazil [76,16]. For this reason, we restricted our analysis to presence/absence data and assumed that once “invaded”, a municipality remains “invaded”, even if no additional cases are detected. Second, the aggregate nature of our data (i.e., the smallest unit of analysis was the municipality) may have masked associations with other factors (e.g., the influence of socio-economic conditions on the presence of the vector and infected hosts).

We used a Bayesian model to better define the risk factors for the spread of VL in São Paulo State, integrating data on its vector and hosts, and to predict the distribution and dispersion of the disease over time and space. As we already cited above, zoonotic VL has been spreading in other regions of Brazil outside of São Paulo [77,24,38] and in other countries as well [21]. Extension of our model to other scenarios can offer further insights into the factors influencing the spread of leishmaniasis and other diseases elsewhere.

## Conclusion

Landscape features, such as the gas pipeline and Marechal Rondon Highway and annual temperature, rather than economic factors, were the most important risk factors in predicting VL dispersion in São Paulo State, Brazil. Since the dispersion of infected humans is affected by the spatial distribution of vectors and infected dogs, strategies to block the spread of the disease in humans and dogs need to address all three components of the VL dynamic cycle. Prevention and control measures should not only focus on vector control (e.g., use of residual insecticide), but also employ measures to block vector-human contact (e.g., insecticide spraying) and vector-dog contact (e.g., insecticide impregnated collars and vaccines). We suggest that these measures be prioritized for areas that have no current record of vectors or hosts infected with *L*. *i*. *chagasi*, but display invasion potential based on our predictions. Our study represents the first published investigation of the interdependent processes involved in the dispersion of VL competent vectors, infected dogs, and infected humans in São Paulo State and the associated risk factors, showing and anticipating the relentless progressive spread of VL. The prediction of VL dispersion is crucial for the identification of areas of high risk and to enable more spatially targeted prevention and control measures, potentially improving public and animal health policy-making.

## Supporting information

S1 Fig Average Gross Domestic Product (*GDP*) for São Paulo State municipalities, Brazil. Values represent Brazilian currency, Reais (R$), classified according to quantile intervals, rounded to the nearest thousand.
(TIF)

S2 Fig Location of the Marechal Rondon Highway, Bolivia-Brazil gas pipeline and municipality centroids in São Paulo State, Brazil. (TIF)

S3 Fig Altitude variations in São Paulo State municipalities, Brazil. (TIF)

S4 Fig Temperature variations in São Paulo State municipalities, Brazil. (TIF)

S5 Fig Average annual rainfall (mm) variations in São Paulo State municipalities, Brazil. (JPG)

S1 Movie Visceral leishmaniasis vector and infected host dispersion (1999–2020). (MP4)

S1 Appendix Detailed tutorial of the Bayesian model in the R program. (PDF)

S1 Table Top 20 municipalities in terms of invasion probability in 2020 for vectors, infected dogs, and infected humans. Municipality superscript numbers are represented on the map in Fig 2.
(PDF)
