# Supplementary figures and images for: Risk analysis and prediction of visceral leishmaniasis dispersion in São Paulo State, Brazil
Source: PLoS Negl Trop Dis. 2017 Feb 6;11(2):e0005353. doi: 10.1371/journal.pntd.0005353 (PMC5313239; doi:10.1371/journal.pntd.0005353)

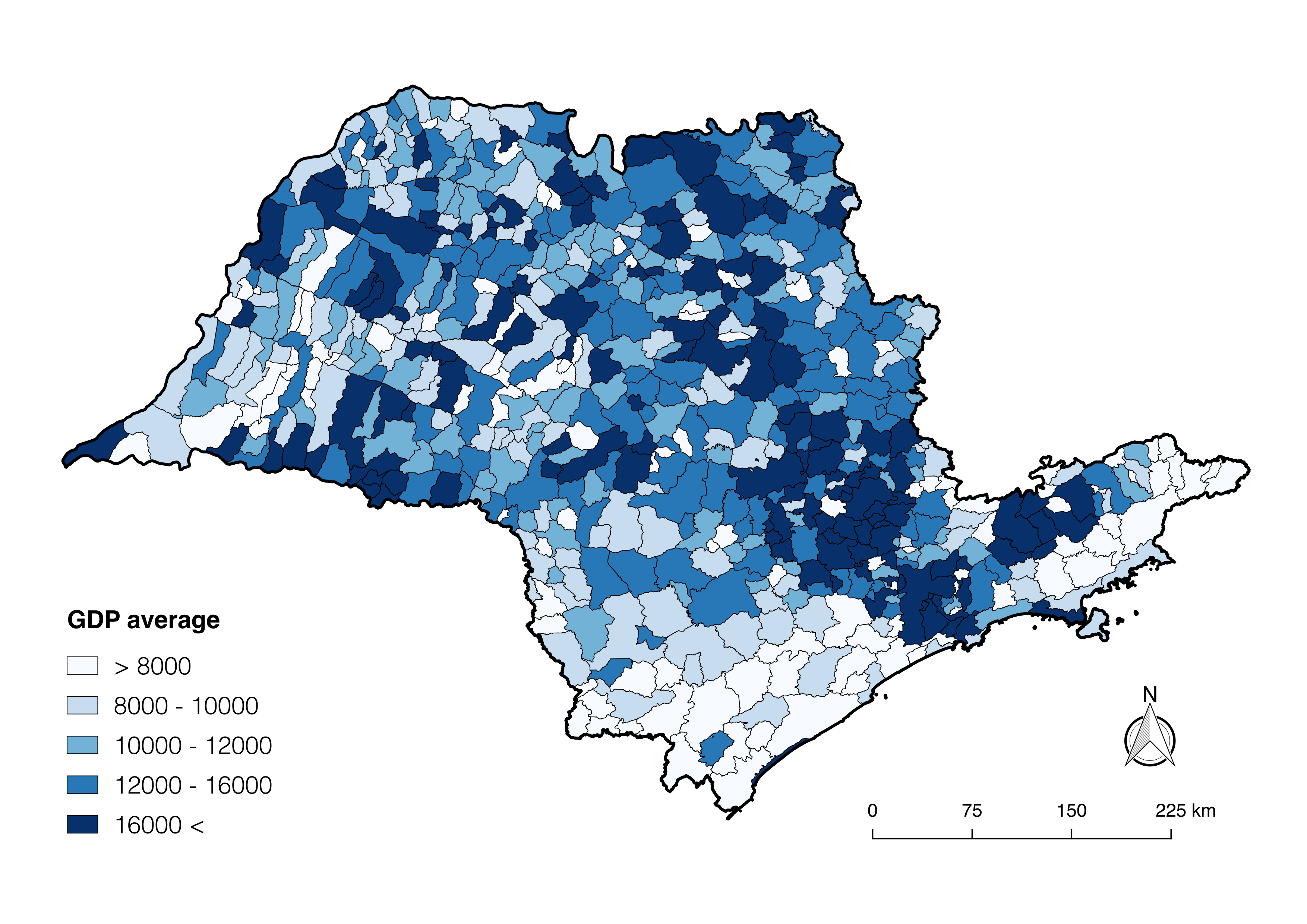

Supplement: S1 Fig — Values represent Brazilian currency, Reais (R$), classified according to quantile intervals, rounded to the nearest thousand. (TIF) [file pntd.0005353.s001.tif]

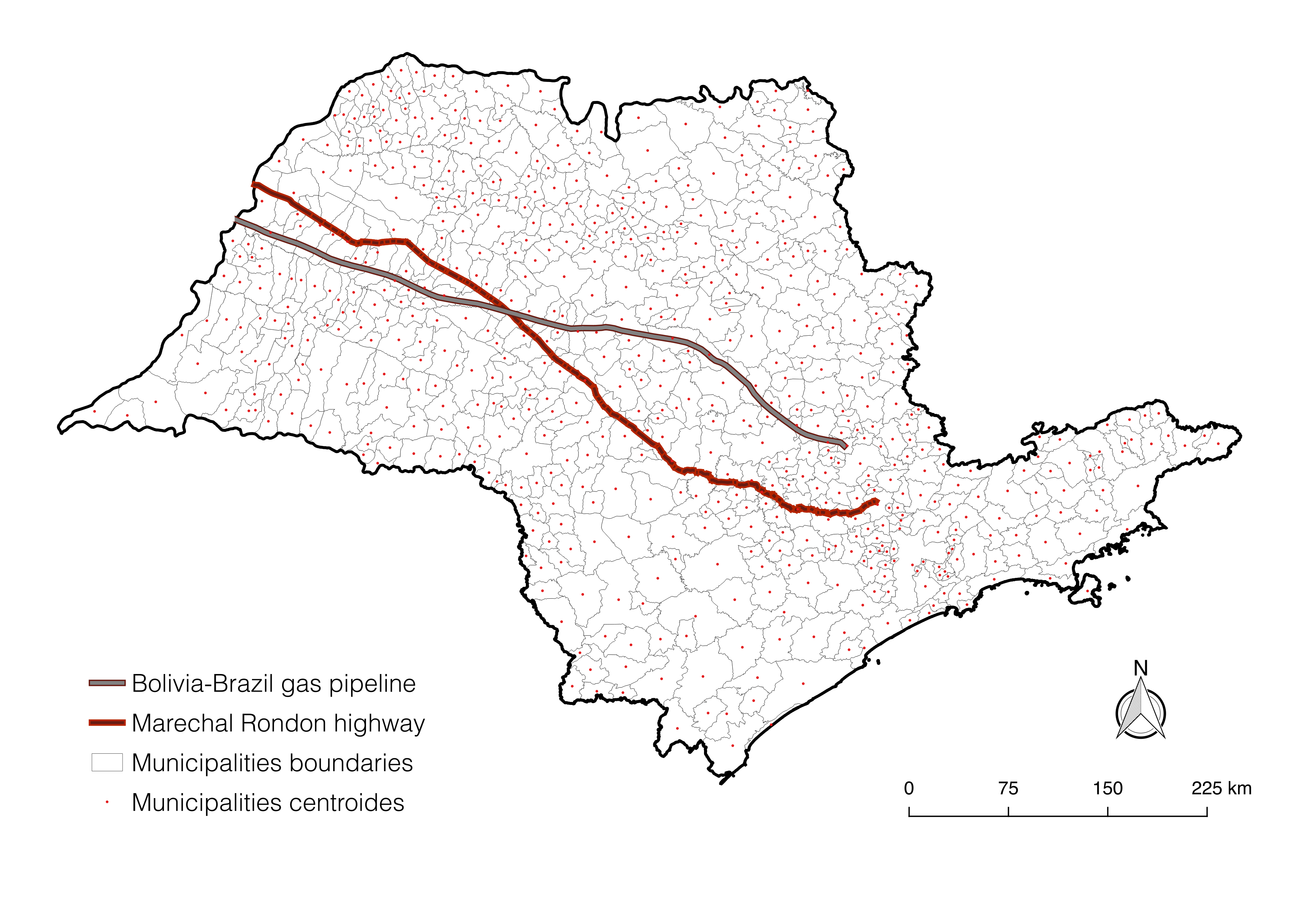

Supplement: S2 Fig — (TIF) [file pntd.0005353.s002.tif]

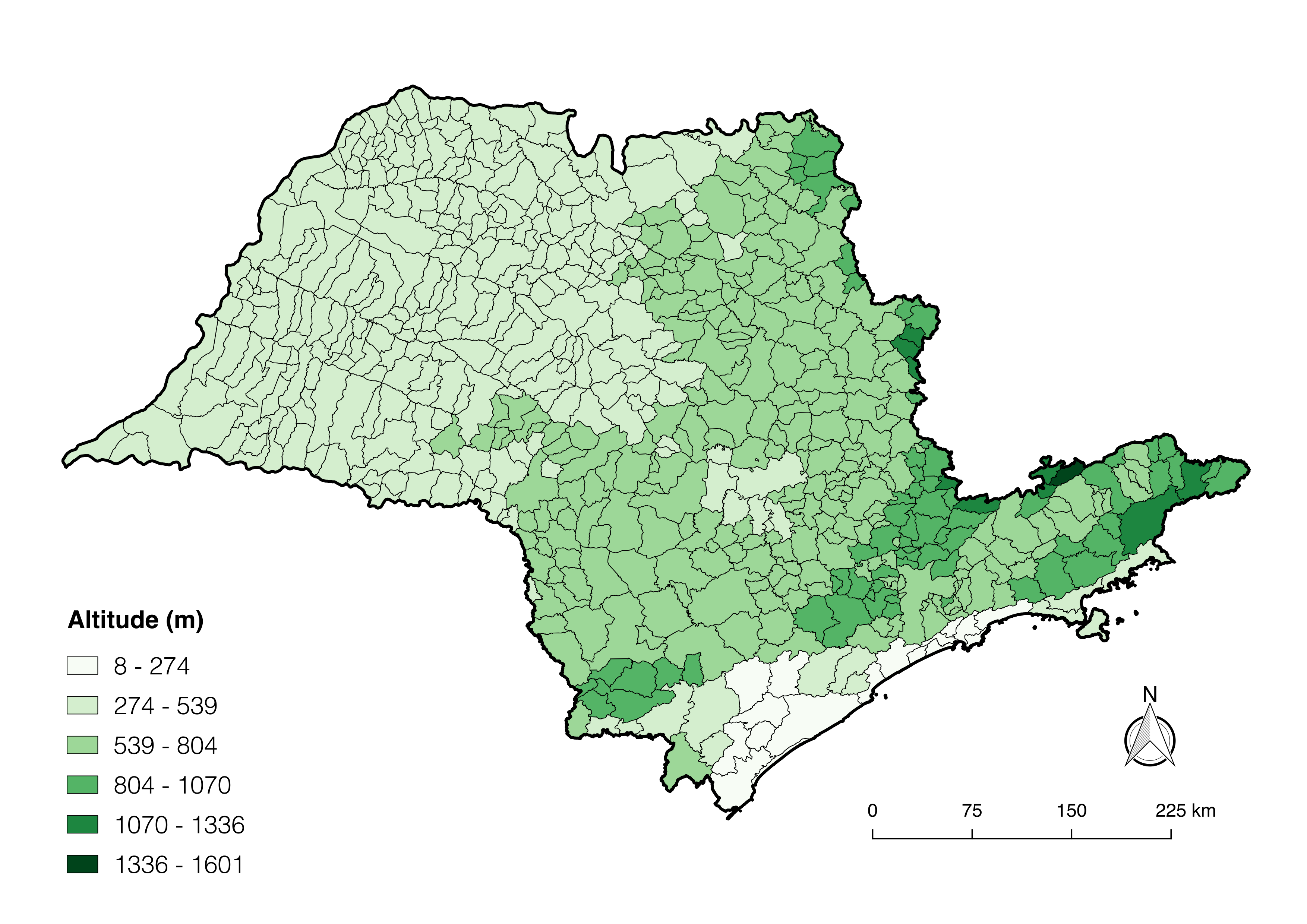

Supplement: S3 Fig — (TIF) [file pntd.0005353.s003.tif]

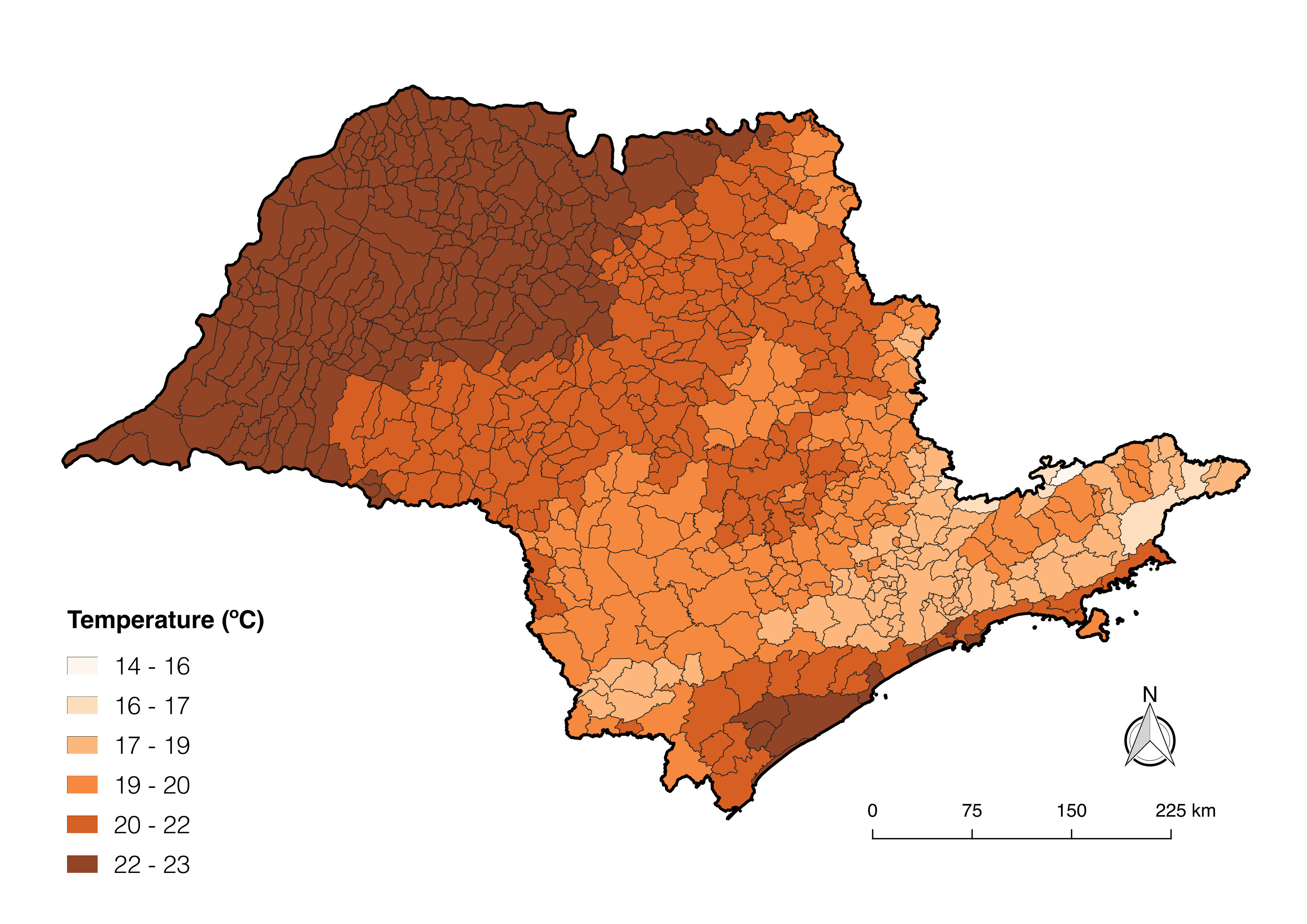

Supplement: S4 Fig — (TIF) [file pntd.0005353.s004.tif]

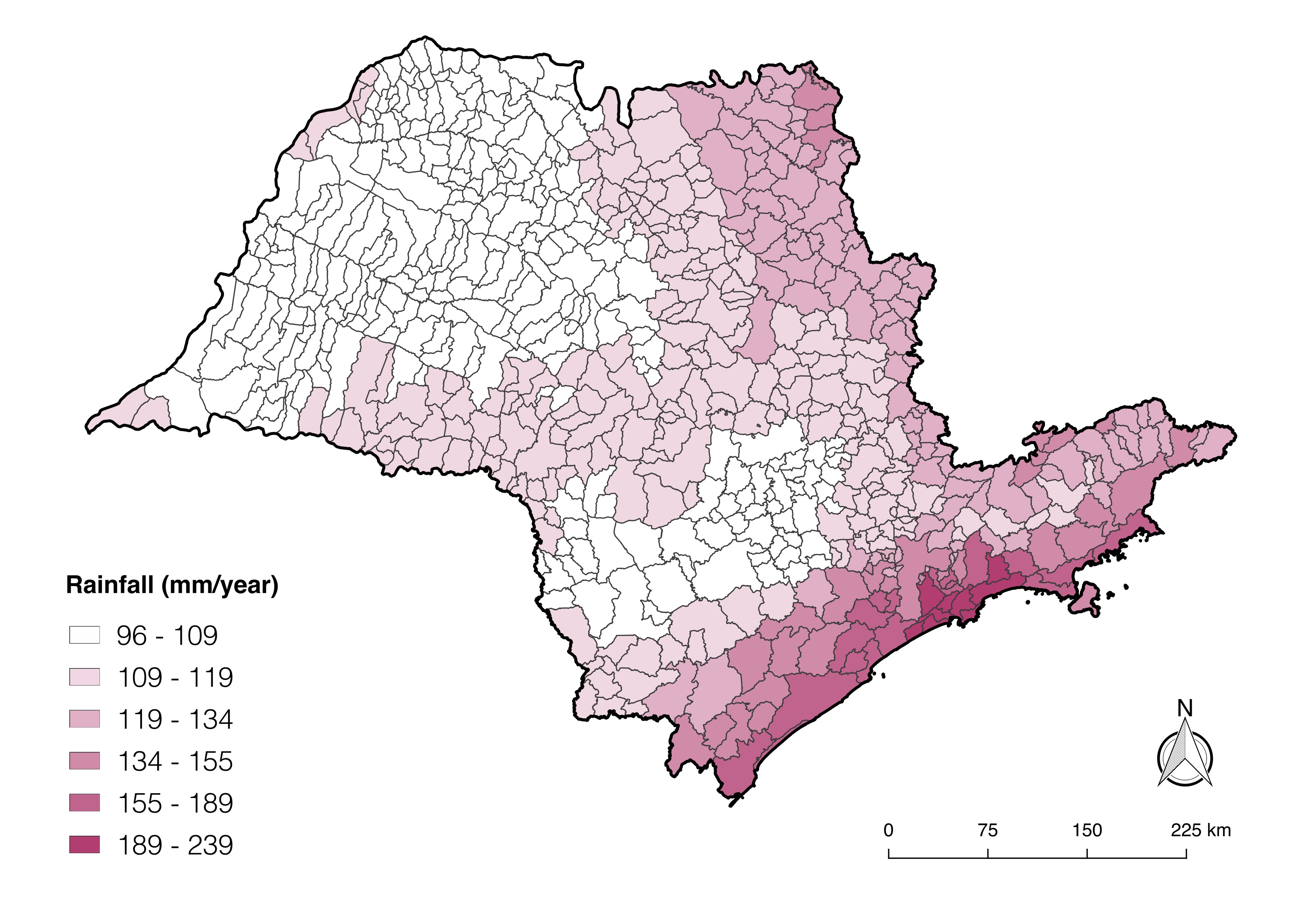

Supplement: S5 Fig — (JPG) [file pntd.0005353.s005.jpg]
