# Supplementary material for: Risk analysis and prediction of visceral leishmaniasis dispersion in São Paulo State, Brazil
Source: PLoS Negl Trop Dis. 2017 Feb 6;11(2):e0005353. doi: 10.1371/journal.pntd.0005353 (PMC5313239; doi:10.1371/journal.pntd.0005353)

Detailed tutorial-style of the Bayesian model in R program. Correspondent to the paper: "Risk analysis and prediction of visceral leishmaniasis dispersion in São Paulo State, Brazil"

Generating simulated data

We start by showing how to create simulated data. These data will then be useful to determine if our code is working as expected.

```
rm(list=ls(all=TRUE))
set.seed(10)

#get covariates
cov=read.csv('covariates standardized.csv',as.is=T)
rodis=cov$rod.dist
gadis=cov$gas.dist
alt=cov$Altitude
tem=cov$Temp
plu=cov$log.rain
pib=cov$log.pib

#get distance matrix
distmat=as.matrix(dist(cov[,c('x','y')]))/1000

#get basic parameters of the simulation
nyear=15
nloc=645

#true regression parameters
param=c(-1,3,2,3,-1,1,1,-1)

#create the invasion status of each Municipality for each year
#(rows are locations, columns are years)
s=matrix(0,nloc,nyear)
s[1,]=1 #assumes that only the first Municipality was invaded in year 1

options(warn=2)
  for (i in 2:nyear){
   for (j in 1:nloc){
   print(c(i,j))
    if (s[j,i-1]==0){ #not occupied yet
     #calculate invasion pressure index
     weight=(1/distmat[j,])
          weight[!is.finite(weight)]=0
          weight1=weight/sum(weight)
          prev=sum(weight1*s[,i-1])
     #calculate probability of invasion
     tmp=exp(param[1]+param[2]*prev+param[3]*gadis[j]+param[4]*rodis[j]+
          param[5]*pib[j]+param[6]*alt[j]+param[7]*tem[j]+param[8]*plu[j])
          theta=tmp/(1+tmp)
          k=rbinom(1,size=1,prob=theta)
          if (k==1) s[j,i:nyear]=1  #once invaded, always invaded
      }
    }
  }
#output fake invasion data
write.csv(s,'fake data.csv',row.names=F)
```

Fitting these data using JAGS

To fit these data using JAGS, we will need to specify our statistical model. We will do this within the file 'vetor.bug'. Here is the content of this file:

```
model{
    for (i in 1:nloc){
        for (j in 2:ntime){
            tmp[i,j] <- exp(b0+b1*pinf[i,j]+b2*gadis[i]+b3*rodis[i]+b4*pib[i]+b5*alt[i]+
                                b6*tem[i]+b7*plu[i])
            tmp1[i,j] <- tmp[i,j]/(1+tmp[i,j])
            prob[i,j] <- pow(tmp1[i,j],auxiliar[i,j])
            obs[i,j]~dbern(prob[i,j])
        }
    }

b0 ~ dnorm(0,1/10)
b1 ~ dnorm(0,1)
b2 ~ dnorm(0,1)
b3 ~ dnorm(0,1)
b4 ~ dnorm(0,1)
b5 ~ dnorm(0,1)
b6 ~ dnorm(0,1)
b7 ~ dnorm(0,1)

}
```

Notice that this model specification requires information on the covariates:

- invasion pressure ('pinf'),
- distance to gas pipeline ('gadis')
- distance to highway ('rodis'),
- gross domestic product ('pib'),
- altitude ('alt'),
- temperature ('tem'),
- rainfall ('plu')

We also require a matrix called 'auxiliar'. This matrix holds the value of 1 if that particular Municipality at that particular time has not been invaded yet or has just been invaded, otherwise assuming the value of 0. We generate this matrix and invoke this model from within R. Here is the code for this:

```
rm(list=ls(all=T))
library('rjags')
source('functions JAGS.R')

#get response variable
obs=read.csv('fake data.csv',as.is=T)

#get covariates
cov=read.csv('covariates standardized.csv',as.is=T)

rodis=cov$rod.dist
gadis=cov$gas.dist
alt=cov$Altitude
tem=cov$Temp
```

```
plu=cov$log.rain
pib=cov$log.pib

#get distance matrix
distmat1=as.matrix(dist(cov[,c('x','y')]))/1000
nloc=nrow(distmat1)

#get auxiliar matrix
ntime=ncol(obs)
auxiliar=get.auxiliar(obs)

#get invasion pressure index
pinf=get.pinf(obs)

#sets up model object
jags=jags.model('vetor.bug',data=list('obs'=obs,'pinf'=pinf,'nloc'=nloc,
                'ntime'=ntime,'auxiliar'=auxiliar,'gadis'=gadis,
                'rodis'=rodis,'pib'=pib,'alt'=alt,'tem'=tem,
                'plu'=plu),n.chains=4,n.adapt=100)

#burn-in period
update(jags,1000)

#draw samples from the sampler
k=jags.samples(jags,c('b0','b1','b2','b3','b4','b5','b6','b7'),1000)

#output samples from posterior distribution
res=numeric()
for (i in 1:length(k)) res=cbind(res,as.numeric(k[[i]]))
write.csv(res,'parameter estimates.csv',row.names=F)
```

Notice that this code requires two customized functions “get.auxiliar” and “get.pinf”, which are stored in the file “functions JAGS.R”. The code in this file is given below:

```
#calculate invasion pressure index
get.pinf=function(s){
  s1=matrix(0,nrow(s),ncol(s))

  for (i in 2:ncol(s)){
    for (j in 1:nrow(s)){
      #calculate weights
      weights=1/distmat1[j,]
      weights[!is.finite(weights)]=0
      weights1=weights/sum(weights)

      #calculate index
      s1[j,i]=sum(weights1*s[,i-1])
    }
  }
}

#-------------------------------------------
#get auxiliar matrix. This matrix has 1's prior to invasion and in the year of invasion
#otherwise it has a zero
get.auxiliar=function(s){
  auxiliar=matrix(0,nloc,ntime)
```

```
    for (i in 1:nloc){
    ind=which(s[i,]==1)
    if (length(ind)==0) auxiliar[i,]=1 #never invaded
    if (length(ind)==ntime) auxiliar[i,]=0 #always invaded
    if (length(ind)>0 & length(ind)<ntime) auxiliar[i,1:min(ind)]=1 #invaded in
between
  }
  auxiliar
}
```

We check if the algorithm is working by comparing its outputs to the true parameter values used to create this fake data. These results suggest that our algorithm works well:

```
#compare estimated parameters to the true parameter values
param.estim=read.csv('parameter estimates.csv',as.is=T)
param.true=c(-1,3,2,3,-1,1,1,-1)

par(mfrow=c(1,1),mar=rep(4,4))
param.estim1=apply(param.estim,2,mean)

range1=range(c(param.estim1,param.true))
plot(param.true,param.estim1,xlim=range1,ylim=range1,xlab='True parameters'
      ,ylab='Estimated parameters')
lines(range1,range1,col='grey')

#get 95% credible intervals
cred.interv=apply(param.estim,2,quantile,c(0.025,0.975))
for (i in 1:length(param.true)) lines(rep(param.true[i],2),cred.interv[,i])
```

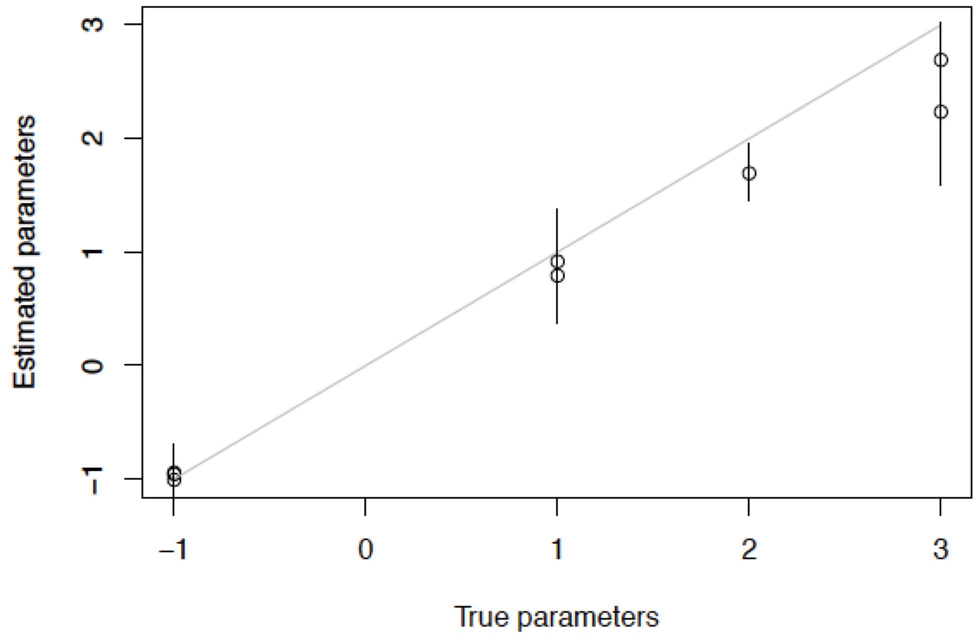

Fitting these data using a standard logistic regression

These data can also be fit using a standard logistic regression. However, to do this, the data have to be stacked such that there is a single column with the response variable with the other columns containing the covariate information. Furthermore, these data should only include results for non-invaded municipalities (i.e., exposed municipalities) and for those municipalities that have just been invaded.

```
rm(list=ls(all=T))
source('functions JAGS.R')

#get response variable
obs=read.csv('fake data.csv',as.is=T)

#get covariates
cov=read.csv('covariates standardized.csv',as.is=T)

rodis=cov$rod.dist
gadis=cov$gas.dist
alt=cov$Altitude
tem=cov$Temp
plu=cov$log.rain
pib=cov$log.pib

#calculate distance matrix
distmat1=as.matrix(dist(cov[,c('x','y')]))/1000
nloc=nrow(distmat1)

#get response variable
ntime=ncol(obs)
auxiliar=get.auxiliar(obs) #get auxiliar matrix
pinf=get.pinf(obs) #get invasion pressure index

#re-format the data so that we only keep results for non-invaded sites
#and for sites that have just been invaded
obs1=obs
dados=numeric()
for (i in 2:ncol(obs1)){
   ind=which(obs1[,i-1]==0)
   tmp=cbind(obs1[ind,i],pinf[ind,i],cov[ind,c('gas.dist','rod.dist',
                              'log.pib','Altitude','Temp','log.rain')])
   dados=rbind(dados,tmp)
}

colnames(dados)[1:2]=c('just.invaded','prop.infected')

#run GLM
z=glm(just.invaded~prop.infected+gas.dist+rod.dist+log.pib+
                  Altitude+Temp+log.rain,family='binomial',data=dados)

#compare true to estimated parameters
param.true=c(-1,3,2,3,-1,1,1,-1)
range1=range(c(z$coefficients,param.true))
plot(param.true,z$coefficients,xlim=range1,ylim=range1,xlab='True
parameters',
      ylab='Estimated parameters')
lines(range1,range1,col='grey')

#get 95% confidence intervals
```

```
z1=summary(z)
z2=rbind(z1$coefficients[,1]-2*z1$coefficients[,2],
      z1$coefficients[,1]+2*z1$coefficients[,2])
for (i in 1:length(param.true)) lines(rep(param.true[i],2),z2[,i])
```

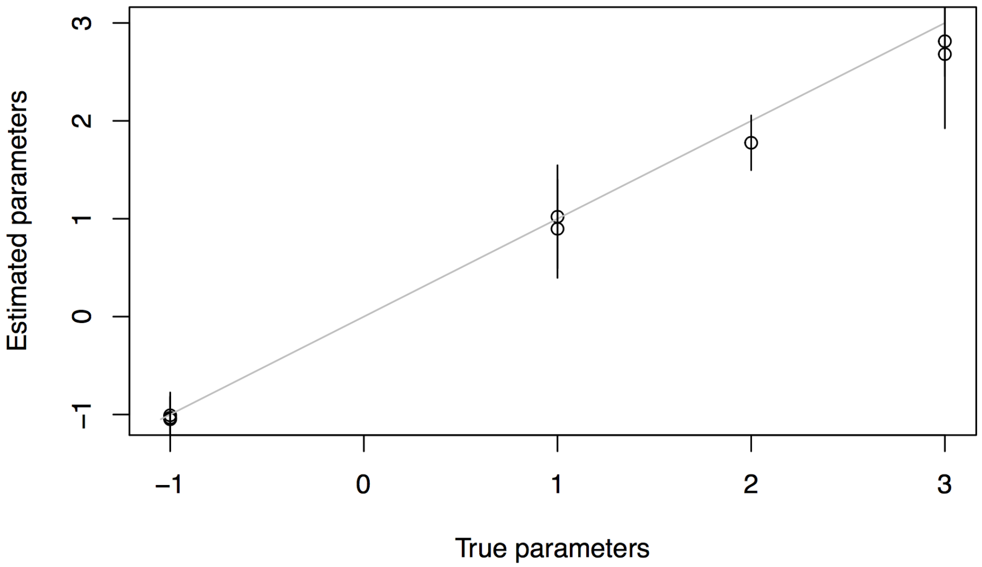

Supplement: S1 Appendix — (PDF) [file pntd.0005353.s007.pdf]
