# Supplementary material for: Risk analysis and prediction of visceral leishmaniasis dispersion in São Paulo State, Brazil
Source: PLoS Negl Trop Dis. 2017 Feb 6;11(2):e0005353. doi: 10.1371/journal.pntd.0005353 (PMC5313239; doi:10.1371/journal.pntd.0005353)
Supplement: S1 Table — Municipality superscript numbers are represented on the map in Fig 2. (PDF) [file pntd.0005353.s008.pdf]

	VECTORS	DOGS	HUMANS
1º	Emilianópolis[9]	Balbinos[5]	Luizinia[23]
2º	Ribeiro dos Índios[38]	Sabino[39]	Alto Alegre[3]
3º	Zacarias[49]	Guaimbê[14]	Santópolis do Aguapeí[42]
4º	Queiroz[35]	Arco-Íris[4]	Glicério[13]
5º	Reginópolis[37]	Iacri[16]	Lourdes[22]
6º	Gastão Vidigal[11]	Pracinha[34]	Buritama[7]
7º	Ponga[33]	Herculândia[15]	Alfredo Marcondes[2]
8º	Ubarana[47]	Parapuã[30]	Sud Mennucci[45]
9º	Adolfo[1]	Santo Expedito[41]	Gabriel Monteiro[10]
10º	Nova Castilho[27]	Nova Canaã Paulista[26]	Brejo Alegre[6]
11º	Júlio Mesquita[20]	Nova Luzitânia[28]	Itapura[19]
12º	Sales[40]	Marinópolis[24]	Palmeira d'Oeste[29]
13º	General Salgado[12]	Ipena[18]	Balbinos[5]
14º	Uru[48]	Reginópolis[37]	Guaimbê[14]
15º	São João das Duas Pontes[43]	Pongaí[33]	Presidente Alves[52]
16º	São João de Iracema[44]	Pompéia[32]	Pracinha[34]
17º	Planalto[31]	Quintana[36]	Sabino[39]
18º	Mendonça[25]	Caiabu[8]	Suzanópolis[55]
19º	José Bonifácio[21]	Três Fronteiras[46]	Meridiano[51]
20º	Indiana[17]	Queiroz[35]	Arealva[50]<br>Santana da Ponte Pensa[53]
